# Complete chloroplast genome sequence of *Betula microphylla* (Betulaceae)

**DOI:** 10.1080/23802359.2020.1768923

**Published:** 2020-05-23

**Authors:** Donglai Hua, Jianbo Zhu

**Affiliations:** a College of Life Sciences, University of Shihezi, Shihezi, China;; b Institute of Botany, Chinese Academy of Sciences, Beijing, China;; c Xinjiang Academy of Agricultural and Reclamation Science, Shihezi, China

**Keywords:** *Betula microphylla*, Betulaceae, complete chloroplast genome, phylogenetic tree

## Abstract

*Betula microphylla* (Betulaceae) is a species widely distributed in Xinjiang China and in Mongolia and Siberia of Russia. In this study, we described the complete chloroplast genome of *B. microphylla* based on Illumina paired-end sequencing. The chloroplast genome of *B. microphylla* is 160,489 bp long, including two inverted repeats (IRs, 26,070 bp), separated by a large single-copy region (LSC, 89,306 bp), and a small single-copy region (SSC, 19,045 bp). The overall GC content of the whole genome is 36.1%, and the corresponding values of the LSC, SSC, and IR regions are 33.7%, 29.7%, and 42.5%. The phylogenetic analysis indicated that *B. microphylla* is closely related to *Betula occidentalis*.

*Betula microphylla* Bunge was described from the Altai Mountains. Distributed in the Altai Mountains and Xinjiang and neighboring Kazakstan. The dry leaves of *B. microphylla* were collected from Xinjiang Altai mountains（47°45′21.6″N, 85°46′38.8″E）. The voucher specimen was deposited at the Herbarium of the Institute of Bontany, the Chinese Academy of Sciences (PE) (accession number CPG28261). The DNA was sheared to 500 bp and prepared in the DNA library. Total 2.15 Gb 100-bp pair-end reads were generated on Illumina HiSeq 2500 (Illumina, San Diego, USA). The raw reads were filtered for low-quality bases (PHRED < 20) by NGSQC Toolkit v2.3.3 ((Patel and Jain 2012). De novo assembly was conducted in VELVET v1.2.10 (Zerbino and Birney 2008). Plastome sequences were picked up by blasting all contigs against confamilial species *Castanea mollissina* (accession number: NC_014674) using BLAST v2.4.0 (Camacho et al. 2009) with default parameter, then were assembled manually in Geneious R7.19 (Biomatters, Inc., Auckland, New Zealand; http://www.geneious.com). Gene annotation was determined by DOGMA (Wyman et al. 2004) and PGA(Qu et al. 2019), and adjusted manually in Geneious R7.19. The genome sequence has been submitted to NCBI (https://www.ncbi.nlm.nih.gov/) under accession number MT310900.

To confirm the phylogenetic position of *B. microphylla*, other eight species of genus Betula from NCBI were aligned using MAFFT v.7 (Katoh and Standley 2013) and maximum-likelihood (ML) bootstrap analysis was conducted using RAxML (Stamatakis et al. 2008; Stamatakis 2014); bootstrap probability values were calculated from 1000 replicates. *Carpinus putoensis* (NC_033503) were served as the out-group.

The complete *B. microphylla* plastid genome is a circular DNA molecule with the length of 160,489 bp, contains a large single-copy region (LSC) of 89,306 bp and a small single-copy region (SSC) of 19,045 bp, which were separated by a pair of inverted repeat (IR) regions of 26,070 bp. The overall GC content of the whole genome is 36.1%, and the corresponding values of the LSC, SSC, and IR regions are 33.7%, 29.7%, and 42.5%, respectively. The plastid genome contained 132 genes, including 84 protein-coding genes, eight ribosomal RNA genes, and 42 transfer RNA genes. Phylogenetic analysis showed that *B. microphylla* clustered in a unique clade in genus *Betula* (Figure 1). The determination of the complete plastid genome sequences provided new molecular data to illuminate the Betula evolution. The complete chloroplast of *B. microphylla* would represent a useful genetic resource to the further biological and evolutionary studies.

**Figure 1.**
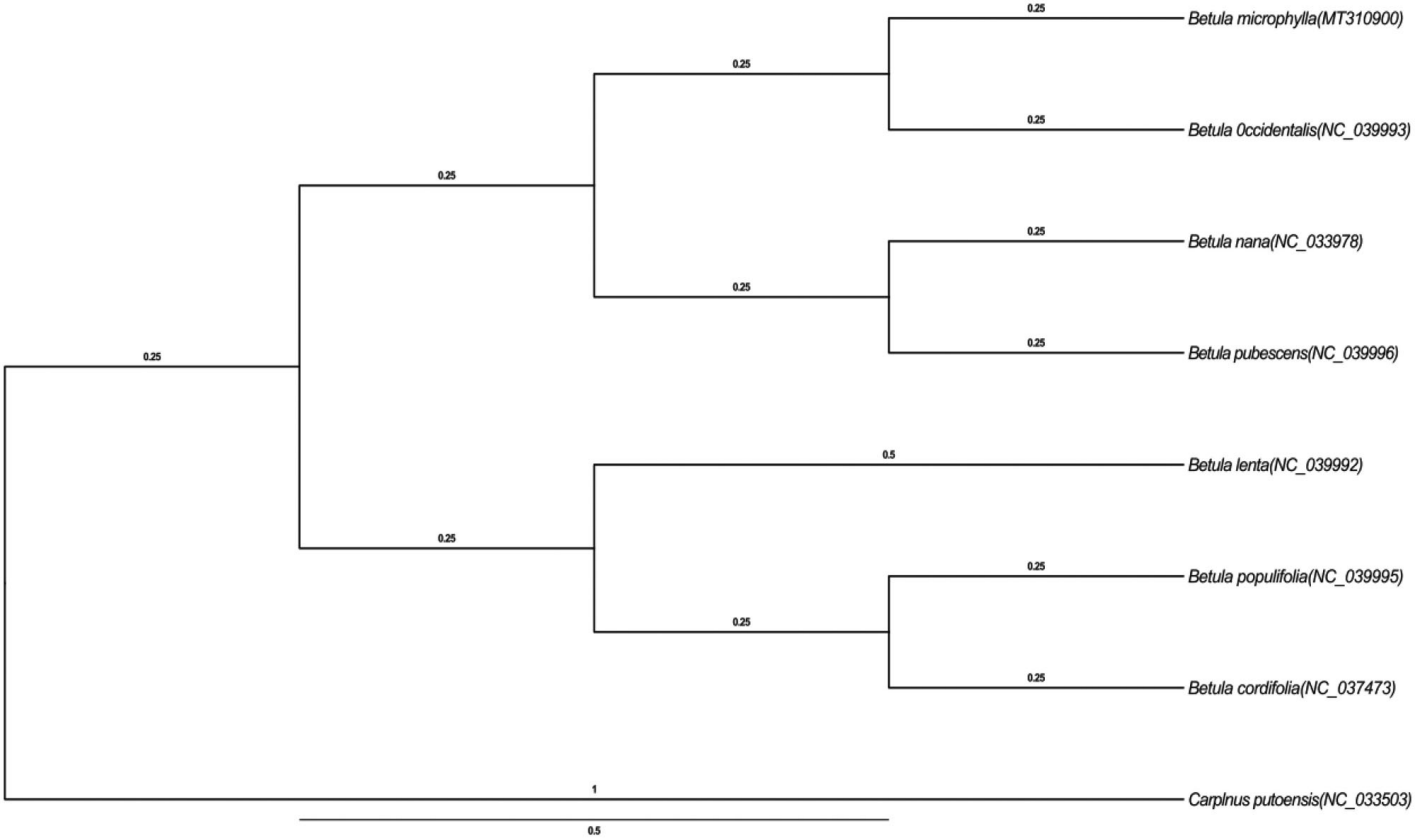
Phylogenetic tree based on the chloroplast genomes of eight plastomes. Bootstrap support values from 1000 replicates are shown branches. All the plastome sequences are available in GenBank, with the accession numbers listed right next to their scientific names.

## Data Availability

The data that support the findings of this study are openly available in NCBI, GenBank accession number for this study: MT310900. https://www.ncbi.nlm.nih.gov/. Please note data should only be shared if it is ethically correct to do so, where this does not violate the protection of human subjects, or other valid ethical, privacy, or security concerns.
